# Diverse *in vivo* effects of soluble and membrane-bound M-CSF on tumor-associated macrophages in lymphoma xenograft model

**DOI:** 10.18632/oncotarget.6362

**Published:** 2015-11-22

**Authors:** Jinfeng Liao, Wenli Feng, Rong Wang, Shihui Ma, Lina Wang, Xiao Yang, Feifei Yang, Yongmin Lin, Qian Ren, Guoguang Zheng

**Affiliations:** ^1^ State Key Laboratory of Experimental Hematology, Institute of Hematology and Blood Diseases Hospital, Chinese Academy of Medical Sciences and Peking Union Medical College, Tianjin, China; ^2^ Center for Stem Cell Medicine, Chinese Academy of Medical Sciences, Beijing, China

**Keywords:** mM-CSF, sM-CSF, lymphoma, TAMs, subpopulation

## Abstract

Macrophage colony-stimulating factor (M-CSF) is an important cytokine for monocyte/macrophage lineage. Secretory M-CSF (sM-CSF) and membrane-bound M-CSF (mM-CSF) are two major alternative splicing isoforms. The functional diversity of these isoforms in the activation of tumor-associated macrophages (TAMs), especially in lymphoma microenvironment, has not been documented. Here, we studied the effects of M-CSF isoforms on TAMs in xenograft mouse model. More infiltrating TAMs were detected in microenvironment with mM-CSF and sM-CSF. TAMs could be divided into three subpopulations based on their expression of CD206 and Ly6C. While sM-CSF had greater potential to recruit and induce differentiation of TAMs and TAM subpopulations, mM-CSF had greater potential to induce proliferation of TAMs and TAM subpopulations. Though both isoforms educated TAMs and TAM subpopulations to M2-like macrophages, mM-CSF and sM-CSF induced different spectrums of phenotype-associated genes in TAMs and TAM subpopulations. These results suggested the diverse effects of M-CSF isoforms on the activation of TAMs and TAM subpopulations in lymphoma microenvironments.

## INTRODUCTION

Macrophage colony-stimulating factor (M-CSF), also known as colony-stimulating factor-1 (CSF-1), is the key regulator for monocyte / macrophage lineage [1]. By alternative splicing from a single gene, three biologically active isoforms, *i.e.* secretory (sM-CSF), membrane-bound (mM-CSF) and extracellular matrix or proteoglycan (PG-M-CSF), have been identified [2]. They bind the same receptor, M-CSFR. PG-M-CSF was suggested as an extracellular matrix storage form of sM-CSF [3]. sM-CSF regulates cells nearby or in distance by autocrine, paracrine or endocrine mechanisms, whereas mM-CSF regulates physically contact cells by juxtacrine mechanism [4]. Though cleavage is inefficient, mM-CSF can also be cleaved from cell membrane by TNFα converting enzyme [5]. M-CSF isoforms show distinct characteristics in both physiological and pathological processes. For example, transgenic expression of mM-CSF only partly restored M-CSF function in M-CSF-deficient mice [6] and mM-CSF was more effective to accumulate and activate macrophages in renal inflammation kidney [7]. mM-CSF and PG-M-CSF each shifted the circulating monocyte population toward an inflammatory, activated phenotype more readily recruited to the kidney during lupus nephritis [8].

M-CSF takes part in the pathological process of tumors. High level of M-CSF was detected in breast cancer, ovarian cancer, endometrial carcinoma, and cervical cancer [9, 10]. Furthermore, high sM-CSF level was associated with poor prognosis in colorectal and breast cancers [11, 12]. Abnormal high serum M-CSF level was also reported in pre-leukemia, leukemia, lymphoid malignancies [13] and high level of membrane associated M-CSF was reported in Hodgkin's lymphoma, leukemia and myelodysplastic syndromes (MDS) [14].

Macrophages are essential cellular components of the host defense system and play important roles in both physiological and pathological processes [15]. They have remarkable plasticity, and their functional phenotype is controlled by microenvironmental signals [16–18]. Typically, macrophages can be polarized into two functionally distinct forms representing two extreme phenotypes, *i.e.* M1 and M2 macrophages. As important components of the tumor microenvironments, tumor-associated macrophages (TAMs) promote the progression of tumors in most cases by enhancing angiogenesis, stimulating proliferation, migration and invasion of tumor cells [19]. Though TAMs are regarded as M2 macrophages, they show diverse phenotypes different from classical M2 phenotype in tumor microenvironments [16]. In facts, TAMs with both M1 and M2 characteristics were detected in tumor tissues [20] though M2 macrophages are suggested to have pro-tumor effects and M1 macrophages are suggested to have anti-tumor effects [21]. Meanwhile, subpopulations of TAMs were studied to reveal the nature of macrophages in the development of tumors [22–24].

The significance of macrophages in hematopoietic malignancies is mainly discussed in lymphomas. Lymphoma-associated macrophages were proposed in follicular lymphoma, and suggested to be an independent predictor of overall survival [25, 26]. High TAM counts in lymph node biopsy samples were detected in patients with poor-prognostic classic Hodgkin's lymphoma [27] and different groups further discussed the potential use of TAMs as a biomarker for risk stratification in Hodgkin's lymphoma [28–30]. We previously demonstrated that mM-CSF could be a special linker between macrophage and lymphoma cells [31]. However, the effects of M-CSF isoforms in lymphoma microenvironment on macrophages have not been elucidated.

In this study, we studied the characteristics of TAMs in microenvironment with mM-CSF or sM-CSF in a xenograft mice model. We found that TAMs could be divided into three subpopulations based on the expression of CD206 and Ly6C. While mM-CSF was more potent to induce proliferation, sM-CSF was more potent to recruit and induce differentiation of TAMs and TAM subpopulations. Furthermore, mM-CSF and sM-CSF induced different spectrums of phenotype-associated genes though both isoforms educated TAMs to M2-like macrophages phenotypes.

## RESULTS

### Establishment of Namalwa cell lines stably expressing sM-CSF and mM-CSF

**Figure 1:**
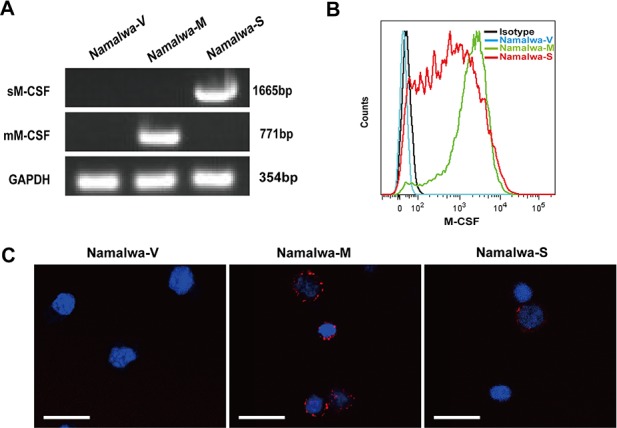
Establishment of Namalwa cell lines stably expressing mM-CSF or sM-CSF Namalwa cells were transfected with mock MSCV-GFP retrovirus or retrovirus carrying mM-CSF or sM-CSF. Stably transfected cell lines (Namalwa-V, Namalwa-M, Namalwa-S) were GFP positive, and obtained by cell sorting. The successful establishment of these cell lines was confirmed by RT-PCR **A.**, flow cytometry **B.**, and confocal microscopy analysis **C.** Scale bars of 20μm are indicated.

To investigate the effects of M-CSF isoforms in tumor microenvironment on macrophages, Namalwa cells, which lack endogenous M-CSF expression, were infected with blank MSCV-GFP retrovirus, retrovirus carrying sM-CSF or mM-CSF. After cell sorting, stably transfected cell lines were named Namalwa-V, Namalwa-S and Namalwa-M, respectively. M-CSF expression was verified by RT-PCR (Figure 1A), flow cytometry (Figure 1B), and confocal microscopy (Figure 1C).

### Distribution of TAMs in xenograft mouse model

**Figure 2:**
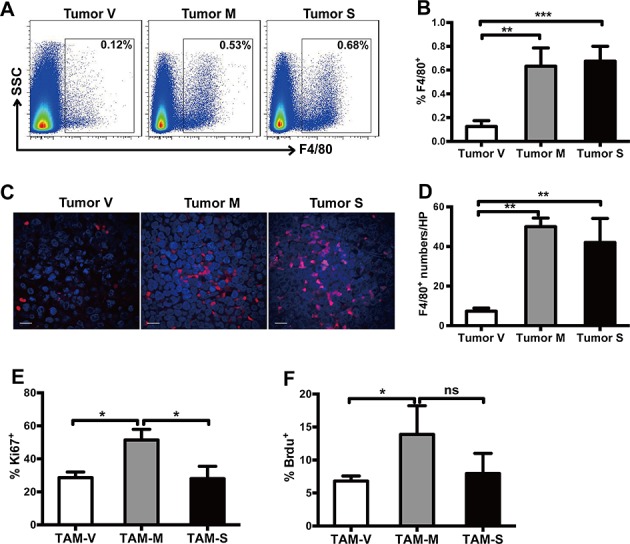
Both mM-CSF and sM-CSF promote infiltration and survival of TAMs **A.**, **B.** Proportion of F4/80^+^ TAMs in tumor tissues was detected by flow cytometry analysis (*n* = 3~5). **C**. Infiltration of F4/80^+^ TAMs in tumor tissues was detected by confocal microscopy analysis. Scale bars of 10μm are indicated. **D**. The number of F4/80^+^ cells in each high power field was counted (*n* = 3). The proportion of Ki67^+^ **E.** and BrdU^+^ **F.** cells in F4/80^+^ TAMs was detected by flow cytometry analysis (*n* = 3). Data in columns are shown as mean ± SD (*, *p* < 0.05; **, *p* < 0.01; ***, *p* < 0.001, ns = not significant).

NOD/SCID mice were subcutaneously implanted with equal number of Namalwa-V, Namalwa-M or Namalwa-S cells. Mice were sacrificed on day 30~35, when the tumor volume was about 1 cm^3^. Flow cytometry analysis showed that the average of infiltrating macrophages was about 0.63% and 0.67%, respectively, in Namalwa-M and Namalwa-S formed tumor tissues (TAM-M and TAM-S, respectively), whereas it was about 0.12% in Namalwa-V formed tumor tissues (TAM-V) (Figure 2A, 2B). This observation was further verified by confocal microscopy analysis (Figure 2C, 2D). These results demonstrated that more TAMs were found in tumor microenvironment with mM-CSF or sM-CSF.

### Proliferation of TAMs

Ki67 staining and BrdU incorporation experiment were used to study the effects of mM-CSF and sM-CSF on the proliferation of TAMs in tumor tissues. 51.4±6.5 % Ki67^+^ TAM-M were detected, whereas only 28.6±3.4% Ki67^+^ TAM-V and 28.0±7.5% Ki67^+^ TAM-S were detected, respectively (Figure 2E). For BrdU incorporation experiment, similar results were observed (Figure 2F). These results indicated that the proliferation potential of TAM-M was significantly higher than that of TAM-V or TAM-S, suggesting that mM-CSF in tumor microenvironment had greater potential to induce proliferation of TAMs.

### Activation phenotype of TAMs

**Figure 3:**
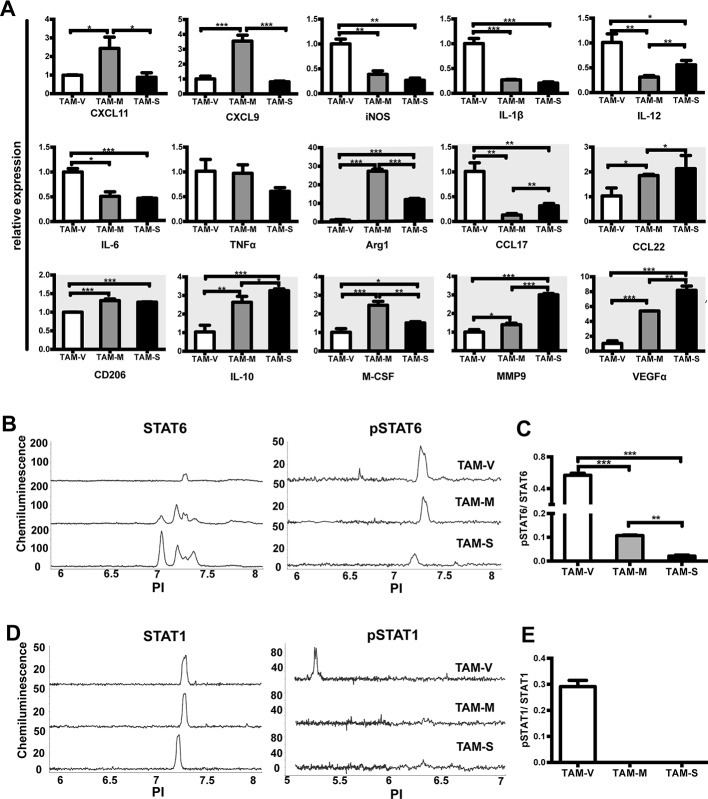
Phenotype of TAMs from different tumor microenvironments Single cell suspension of tumor tissues on Day 30~35 was obtained and TAMs were sorted by flow cytometer after enrichment by magnetic beads. **A**. Expression of phenotype-associated genes in TAMs was detected by real time PCR. For each gene, the RQ value of TAM-V was designated 1.000, respectively. The M2-related genes were shown with dark background. The activation of STAT6 **B.** and STAT1 **D.** signal pathway was studied by Nanopro immunoassay. Peaks represent phosphorylated or unphosphorylated STAT6 and STAT1. The ratio of pSTAT6/STAT6 **C.** or pSTAT1/STAT1 **E.** was calculated by compass software and plotted. Data are shown as mean ± SD (*n* = 3, *, *p* < 0.05; **, *p* < 0.01; ***, *p* < 0.001).

Macrophages have remarkable plasticity, and their functional phenotype is controlled by microenvironmental signals. *In vitro* studies suggested that M-CSF induced monocytes to M2 phenotype macrophages [32]. Here we studied the polarization of macrophages in different microenvironments by analyzing expression of 15 phenotype-associated genes by real time PCR. Compared with TAM-V, TAM-M or TAM-S expressed lower level of most M1-related genes including iNOS, IL-1β, IL-12 and IL-6. In contrast, they expressed higher level of most M2-related genes including Arg1, CCL22, CD206, IL-10, M-CSF, MMP9, and VEGFα (Figure 3A). Moreover, TAM-M and TAM-S showed different spectrums. Significant higher expression of CXCL11, CXCL9, Arg1 and M-CSF was detected in TAM-M, whereas higher expression of IL-12, CCL17, CCL22, IL-10, MMP9 and VEGFα was detected in TAM-S. These data suggested that mM-CSF and sM-CSF had different effects to educate TAMs to M2 phenotypes *in vivo*.

To analyze the systemic effects induced by different Namalwa cells, we also analyzed the expression of the above genes in peritoneal and bone marrow macrophages in the same model (Figure S1). Compared to control group, in bone marrow, macrophages expressed lower level of all M2-related genes. In peritoneal cavity, macrophages expressed lower level of most M2-related genes except Arg1 and CD206. These results indicated that the systemic effects of M-CSF isoforms on peritoneal and bone marrow macrophages were totally different from that on TAMs in tumor microenvironment.

It was reported that macrophage polarization was determined by specific transcription factors. STAT1 is an essential mediator of M1 macrophage polarization, while STAT6 is required to drive M2 macrophage activation [33]. To further investigate the mechanism, total and phosphorylated STAT1 and STAT6 in TAMs were studied by Nanopro immunoassay. The results showed that three TAMs expressed similar level of STAT1 (Figure 3D) while TAM-V expressed lower level of STAT6 (Figure 3B). Furthermore, pSTAT6 was detected in all three TAMs though it was lower in TAM-S (Figure 3B), whereas pSTAT1 was detected in only TAM-V (Figure 3D). Moreover, though lower value of both pSTAT1/STAT1 (Figure 3E) and pSTAT6/STAT6 (Figure 3C) was detected in TAM-M and TAM-S, more drastic decrease was observed in pSTAT1/STAT1 in TAM-M and TAM-S compared to that in TAM-V. Unphosphorylated STATs can also participate in cell signaling [34]. The above results suggested that mM-CSF and sM-CSF in tumor microenvironment polarized TAMs to M2 phenotype by transcriptional regulation.

### Responses of TAMs to LPS and IL-4

**Figure 4:**
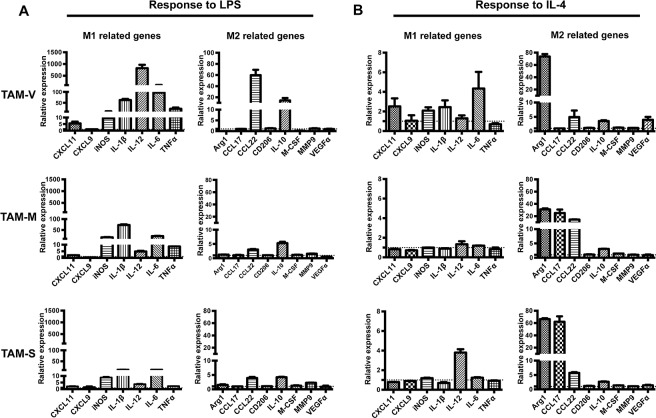
Responses of TAMs to LPS and IL-4 Single cell suspension of tumor tissues on Day 30~35 was obtained and TAMs were sorted by flow cytometer after enrichment by magnetic beads. TAMs were cultured in 24-well plates and treated with LPS **A.** or IL-4 **B.** for 24hrs. The expression of phenotype-associated genes was detected by real time PCR (*n* = 3). For TAMs from different tumor microenvironments, the RQ value of gene expression in untreated TAM-V, TAM-M or TAM-S was designated 1.000, respectively. Data are shown as mean ± SD.

LPS and IL-4 are two stimuli led to the activation phenotype of classical M1 and M2 macrophages [35]. To further investigate the characteristics of TAMs in tumor microenvironment with mM-CSF or sM-CSF, isolated TAMs were stimulated with LPS or IL-4 for 24hrs before real time PCR analysis of phenotype-associated genes. Figure 4 showed the relative fold change of gene expression in TAMs in response to LPS or IL-4 stimulation when untreated TAMs in different groups were set as controls, respectively. Upon LPS stimulation, drastic response was observed in TAM-V, as much higher fold increase was observed in the expression of IL-12, IL-6, TNFα, CCL22, and IL-10 than TAM-M or TAM-S, while decrease in the expression of Arg1 and M-CSF was detected in TAM-V, which was not observed in TAM-M or TAM-S (Figure 4A). The responses of TAM-M and TAM-S were similar though higher fold increase of IL-1β and TNFα could be observed in TAM-M. Upon IL-4 stimulation, high fold increase of Arg1 was observed in all three TAMs though it was higher in TAM-V or TAM-S than TAM-M. Furthermore, different responses could be observed among three TAMs. Higher fold increase of IL-6 and VEGFα was detected in TAM-V; higher fold increase of CCL17 was detected in both TAM-M and TAM-S; higher fold increase of CCL22 was detected in TAM-M whereas higher fold increase of IL-12 was detected in TAM-S. These data suggested that the responses of TAM-M and TAM-S to LPS or IL-4 showed differences to TAM-V. Furthermore, the responses of TAM-M and TAM-S had unique characteristics.

The data were also analyzed when the expression of respective genes in untreated TAM-V was set as control (Figure S2). After LPS stimulation, three TAMs expressed similar level of most genes, except CXCL11, iNOS, M-CSF and VEGFα, which were higher expressed in TAM-M and TAM-S. In contrast, after IL-4 stimulation, TAM-M and TAM-S expressed higher level of most genes except IL-12 and M-CSF, which were similar in three TAMs. These results suggested that TAMs educated by mM-CSF and sM-CSF were more responsive to IL-4, which polarized TAMs to classical M2 phenotype.

### Subpopulations of TAMs

**Figure 5:**
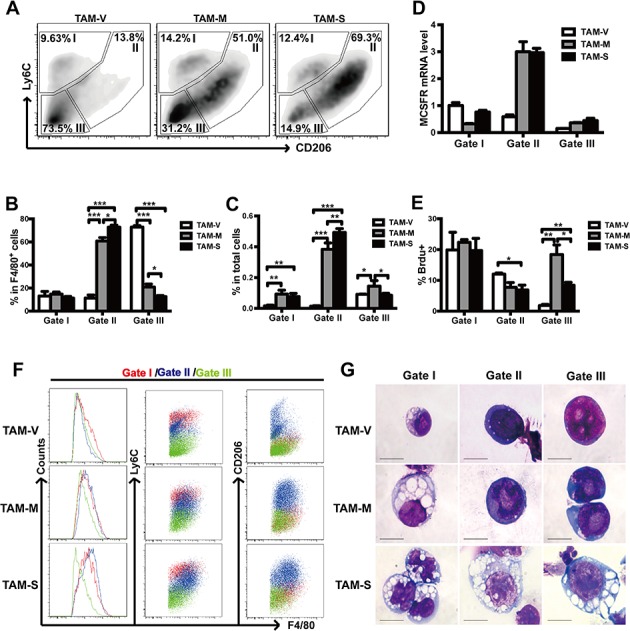
Subpopulations of TAMs from different microenvironments **A.** F4/80^+^ TAMs from different tumor microenvironments were further gated into three subpopulations (gate I, gate II, and gate III) based on the expression of Ly6C and CD206. The percentage of TAMs of different subpopulation was indicated. **B**. Proportion of three TAM subpopulations in F4/80^+^ cells (*n* = 3~5). **C**. Proportion of three TAM subpopulations in total cells of tumor tissues (*n* = 3~5). **D**. Relative expression level of M-CSFR mRNA in three TAM subpopulations. The RQ value of gate I TAM in TAM-V was designated 1.000. **E**. Proportion of BrdU^+^ TAMs in different subpopulations was analyzed by flow cytometry. **F**. Expression of F4/80, Ly6C and CD206 in three TAM subpopulations. **G**. Light microscopic analysis after Wright-Giemsa staining was carried out and typical TAMs are shown. Scale bars of 10μm are indicated. Data in columns are shown as mean ± SD (*n* = 3, *, *p* < 0.05; **, *p* < 0.01; ***, *p* < 0.001).

It has been reported that TAMs were heterogeneous and could be further divided into subpopulations with different biological characteristics [22, 36]. In this study, we labeled TAMs with Ly6C, a surface marker for recruited monocytes and macrophages, and CD206, a surface marker for M2 macrophages. Three obvious TAMs subpopulations were gated and termed gate I, gate II and gate III TAMs (Figure 5A). The majority of TAM-V was gate III TAMs, while the majority of TAM-M and TAM-S was gate II TAMs (Figure 5B). Furthermore, gate I or gate II TAMs in TAM-M and TAM-S were significantly more than those in TAM-V while gate III TAMs in TAM-M were more than those in either TAM-S or TAM-V (Figure 5C). Moreover, gate II and gate III TAMs in TAM-M or TAM-S expressed higher level of F4/80 (Figure 5F), indicating that they were more mature macrophages. Interestingly, just gate II TAMs in TAM-M and TAM-S expressed higher level of the differentiation specific gene M-CSFR (Figure 5D). The typical morphology of TAMs from different gates is shown in Figure 5G. Drastic morphological distinction was observed among TAM-V, TAM-M and TAM-S whereas less distinction was detected among different gates. TAM-S from three gates showed more mature morphology with large cell size and abundant cytoplasm while TAM-M from gate II and gate III were distinguished by their enlarged nucleoli. These data suggested that both mM-CSF and sM-CSF in tumor microenvironment promoted more mature phenotype of TAMs while sM-CSF was more effective.

### Proliferation of TAM subpopulations

The proliferation of TAM subpopulations was studied by BrdU incorporation assay. The results showed that gate I TAMs showed little difference among three groups whereas more BrdU^+^ cells were detected in gate II TAM-V than either TAM-M or TAM-S. It's worth noting that the majority of TAM-M and TAM-S was gate II TAMs (Figure 5A, 5B), which suggested that the increase of gate II TAM-M or TAM-S was mainly due to recruitment rather than proliferation. Furthermore, more BrdU^+^ cells were detected in gate III TAM-M and TAM-S than TAM-V (Figure 5E), which suggested that M-CSF mainly increased gate III TAMs and mM-CSF was more potent than sM-CSF.

### The phenotype of TAM subpopulations

**Figure 6:**
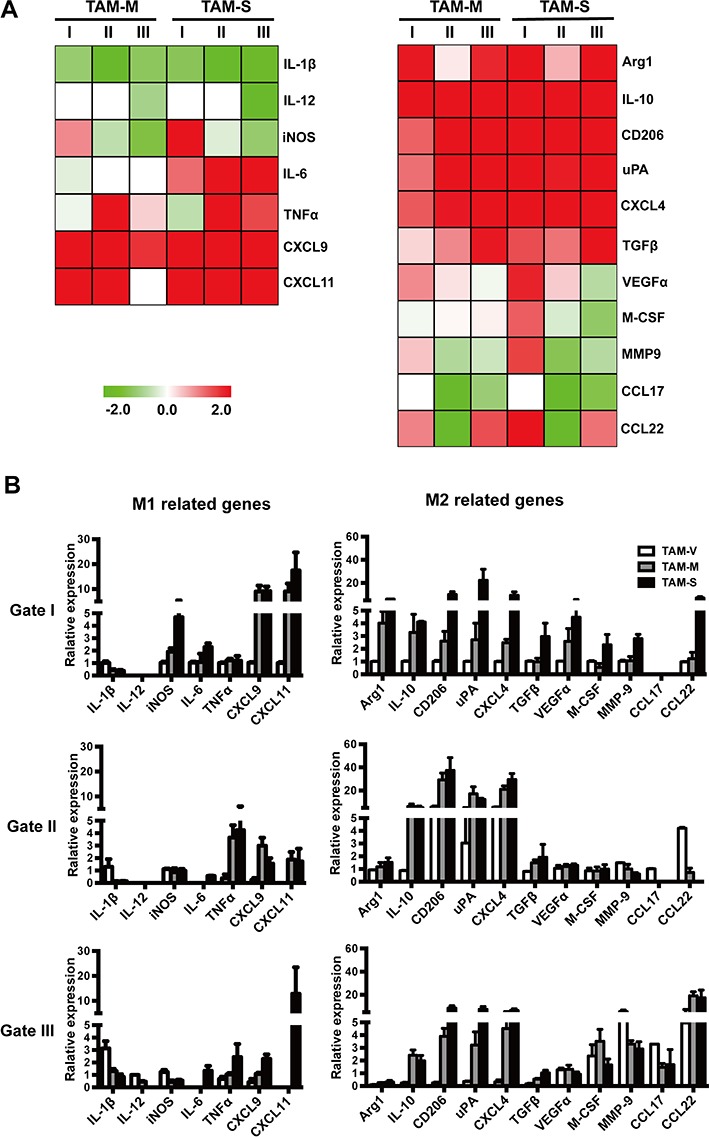
Expression of phenotype-associated genes in different subpopulations of TAMs Ten thousands cells were sorted from each TAM subpopulation, and the expression of phenotype-associated genes was detected by dynamic array analysis. **A**. The heat map shows the expression of phenotype-associated genes in TAM subpopulations of TAM-M and TAM-S normalized to respective TAM subpopulations of TAM-V. **B**. Relative expression level of phenotype-associated genes in TAM subpopulations (*n* = 3). The RQ value of most gene expressions in gate I TAMs in TAM-V was designated 1.000, respectively, except for IL-12 (gate III TAMs in TAM-V was designated 1.000) and CCL17 (gate II TAMs in TAM-V was designated 1.000). Data in columns are shown as mean ± SD.

To further study the characteristics of TAM subpopulations, 10^4^ cells were sorted each gate and dynamic array analysis was performed. Figure 6A plotted the relative expression levels of M1- and M2-related genes in different subpopulations of TAM-M and TAM-S, which were normalized to their respective subpopulations of TAM-V. Compared with subpopulations in TAM-V, TAM-M and TAM-S subpopulations had common features, *i.e.* decreased expression of IL-1β, IL-12 and CCL17 whereas increased expression of CXCL9, CXCL11, Arg1, IL-10, CD206, uPA, CXCL4 and TGFβ, which further confirmed the observation that tumor microenvironment containing both M-CSF isoforms educated TAMs to M2 phenotype. Furthermore, increased expression of iNOS, VEGFα, MMP9 and CCL22 as well as decreased expression of TNFα were detected in gate I in both TAM-M and TAM-S, which were different from the response of gate II and gate III TAMs. It's worth noting that IL-6 was increased significantly in all TAM-S subpopulation, but decreased slightly in all TAM-M subpopulation, which indicated that the two isoforms of M-CSF in tumor microenvironment had different effects on TAMs. The relative expression of these genes in three subpopulations was also analyzed when respective gene expression (except IL-12 and CCL17) of gate I TAMs in TAM-V was designated 1.000 (Figure 6B).

## DISCUSSION

M-CSF is a key regulator for the proliferation, differentiation, activation and function of monocyte/macrophage lineage [37]. Evidence showed that M-CSF isoforms had both shared and unique functions [7, 8]. Until now, to our knowledge, little is known about the diverse effects of M-CSF isoforms, specifically mM-CSF and sM-CSF, on the recruitment, proliferation and activation of TAMs though the effects of sM-CSF on TAMs were reported [38]. In this study, we compared the *in vivo* effects of mM-CSF and sM-CSF on TAMs to explore functional diversity between these isoforms on TAMs in a lymphoma xenograft mouse model.

High level sM-CSF in tumor tissues accounted for the accumulation of TAMs and blockage of CSF1/CSFR signal in tumors could significantly decreased TAMs infiltration [39, 40]. We observed that more TAMs were detected in tumor microenvironment with either mM-CSF or sM-CSF. The rate of recruitment, proliferation and apoptosis determines TAM counts in tumor tissues. sM-CSF was reported to promote macrophage proliferation in tumor microenvironment or in inflammation site [41, 42]. For the first time, we reported that mM-CSF more potently stimulated the proliferation of TAMs than sM-CSF. Furthermore, little difference could be detected on the apoptosis between the two isoforms (6.3±4.6% *VS* 5.4±3.9%, data not shown). Hence, sM-CSF is more potent to recruit TAMs while mM-CSF is more potent to stimulate the proliferation of TAMs. Interestingly, M-CSF isoforms had different effects on the proliferation of TAM subpopulations. Compared with TAM-V, little difference was detected in gate I TAM-M and TAM-S; slight decrease was detected in gate II TAM-M and TAM-S; whereas significant increase was detected in gate III TAM-M and TAM-S. Moreover, mM-CSF was more potent to stimulate the proliferation of gate III TAMs than sM-CSF. It was suggested that mM-CSF could provide stronger and more persistent signals, which accounts for stronger effects of mM-CSF on the proliferation of gate III TAMs, since the membrane integrated mM-CSF hinders the internalization and degradation process [43, 44].

M-CSF affects activation phenotype of macrophages. But the mechanism(s) seem to be complicated. Administration of sM-CSF *in vitro* induced monocytes to M2 phenotype macrophages [18, 32]. However, conflicting results were obtained from *in vivo* studies. Blocking CSF1/CSF1R signaling in mouse pancreatic tumor model preferentially killed M2-like CD206^hi^ TAMs, whereas M1-like CD206^lo^ TAMs were much less affected [45]. However, in spontaneous mammary tumor model, inhibition of CSF1R resulted in similar kinetics for depletion and recovery of both M2-oriented MHC II^lo^ and M1-oriented MHC II^hi^ TAMs [46]. In orthotopic pancreatic tumor model, two CSF1R antagonists significantly deplete macrophages expressing high levels of MHC II, but not MHC II^lo^ or Tie^+^ TAMs [47]. From an overexpression model, we found that M-CSF isoforms in tumor microenvironment induced TAMs with more M2 phenotypes. Analysis of transcription factors further confirmed the observation. Nevertheless, TAM-M and TAM-S showed different expression patterns of phenotype-associated genes and different levels of pSTAT6. These observations suggested that M-CSF isoforms in lymphoma microenvironment polarized macrophages to different states with more M2 phenotypes.

An interesting observation of our results is that TAMs in our model could further be divided into three sub-populations based on the expression of CD206, an M2 macrophage marker, and Ly6C, an important marker for monocytes. Ly6C^+^ and Ly6C^−^ monocytes are regarded as “inflammatory” monocytes and “patrolling or resident” monocytes, respectively [48]. Ly6C^+^ monocytes differentiate into Ly6C^+^ inflammatory macrophages while Ly6C^−^ monocytes differentiate into Ly6C^−^ macrophages [33]. Ly6C^+^ macrophages could also differentiate into Ly6C^−^ macrophages in both tissue repairing model and tumor [22, 49]. TAMs were supposed to be differentiated from both Ly6C^+^ and Ly6C^−^ recruited peripheral blood monocytes [33], but recent evidence suggested that Ly6C^+^ monocytes might be the origin of TAMs [22, 50, 51]. In our model, more gate I and gate II TAMs (Ly6C^+^ subpopulations) were detected while neither high proliferation rates nor low apoptosis rate was detected in both TAM-M and TAM-S. Furthermore, more gate III TAMs (Ly6C^−^ subpopulation) and high proliferation rate were detected in TAM-M. These results imply that both M-CSF isoforms are more potent to recruit Ly6C^+^ monocytes rather than Ly6C^−^ monocytes.

M-CSF isoforms had different effects on phenotypes of TAM subpopulations. Three subpopulations in TAM-S showed more mature morphology with large cell size and abundant cytoplasm despite of differential expressions of F4/80, CD206, Ly6C and M-CSFR. Only gate I TAM-M showed more mature morphology though the majority of TAM-M was detected in gate II which expressed high level of F4/80, CD206 and M-CSFR. Furthermore, analysis of activation-related gene expression also demonstrated that M-CSF isoforms also had different effects on TAM subpopulations despite of similarities.

Taken together, TAMs could be divided into three subpopulations based on the expression of CD206 and Ly6C in a lymphoma xenograft model. More TAMs could be detected in tumor microenvironment with M-CSF isoforms. While mM-CSF had greater potential to induce proliferation of TAMs and TAM subpopulations, sM-CSF had greater potential to recruit and induce differentiation of TAMs and TAM subpopulations. Though both M-CSF isoforms educated TAMs and TAM subpopulations to the states with more M2-like macrophage phenotypes, mM-CSF and sM-CSF induced different spectrums of activation-related genes in TAMs and TAM subpopulations.

## MATERIALS AND METHODS

### Cell lines and antibodies

Human lymphoma cell line Namalwa was obtained from American Type Culture Collection (Manassas, VA). Namalwa cells were infected with MSCV-PGK-GFP retrovirus or recombinant virus carrying mM-CSF or sM-CSF. The GFP^+^ stable transfected cell lines, named Namalwa-V, Namalwa-M or Namalwa-S, were sorted by flow cytometry, respectively. All cells were cultured in RPMI 1640 supplemented with 10% fetal bovine serum (Hyclone, Logan, UT) and antibiotics (Hyclone, Logan, UT) in a humidified atmosphere of 5% CO2 at 37°C. All culture supplies were screened and selected on the basis of being endotoxin free.

Fluorescence-conjugated antibodies against mouse F4/80(APC or PE-conjugated, BM8), CD206 (PerCP-Cy5.5-conjugated, C068C2), Ly6C (PE-Cy7-conjugated, HK1.4), Ki67 (PE-conjugated, 16A8) and IgG (Dylight^TM^ 649-conjugated) were from Biolegend (San Diego). Antibodies against human M-CSF, mouse F4/80 were from Abcam (Cambridge, MA); antibodies against mouse STAT1 and pSTAT1 were from cell signaling technology (Beverly, MA); antibodies against mouse STAT6 and pATAT6 were from Santa Cruz (CA).

### Xenograft mouse model

4- to 5-week old female NOD/SCID mice were purchased from Center for Experimental Animals, the Academy of Military Medical Sciences, and housed in the sterile microisolators in the Animal Centre of the Institute of Hematology & Blood Diseases Hospital, CAMS & PUMC. Experimental procedures performed on the mice were approved by the Animal Care and Use Committee at the institutions involved in this study. After irradiated by ^137^Cs with 250 cGy, mice were injected *s.c.* on the dorsal side with 5×10^7^ cells in a volume of 200μL. Mice were sacrificed when the tumor volume was about 1cm^3^ (volume = length×width×width/2).

### Preparation of tumor tissue samples

Mice were sacrificed by cervical dislocation, and tumor tissues were isolated by blunt dissection and grinded into the Petri dish with PBS buffer with EDTA. Then cell suspension was filtered through graded nylon filter and red cells were removed using erythrocyte lysis buffer (8.26 g/L NH4Cl, 1 g/L KHCO3, and 0.037 g/LEDTA, pH 7.35). After washing, enrichment of macrophages and labeling of macrophage-associated antigens were performed. Briefly, CD11b^+^ cells were enriched by anti-CD11b conjugated magnetic microbeads (Miltenyi Biotech, Auburn, CA) following manufacturer's protocol. Then, enriched cells were resuspended in PBS containing 1%FBS and stained with fluorescence-conjugated antibodies. For sorting or analysis of TAMs, cells were stained with APC-conjugated anti mouse F4/80, whereas for sorting or analysis of TAM subpopulations, cells were stained with APC-conjugated anti mouse F4/80, PerCP-Cy5.5-conjugated CD206, PE-Cy7-conjugated Ly6C. PE-conjugated anti mouse F4/80 was used for BrdU incorporation assay.

### FACS analysis and cell sorting

An LSR II cytometer (BD Biosciences, San Jose, CA) was used for FACS analysis and a FACS Aria III (BD Biosciences, San Jose, CA) was used for cell sorting. Flowjo software (TreeStar, San Carlos, CA) was used for data analysis. Standard protocols were followed for all experiments. TAMs in tumor tissues and macrophage in peritoneal cavity were gated as F4/80^+^ population. Macrophages in bone marrow were gated as described previously [20].

### Cell proliferation assay

For BrdU incorporation assay, tumor-bearing mice were injected *i.p.* with 100ul of 10 mg/ml BrdU 16hrs prior to preparation of tumor tissue samples. Then enriched and labeled cells were further stained with BD Pharmingen^TM^ APC BrdU Flow Kit (BD, San Jose, CA) according to manufacturer's instructions. For Ki67 staining assay, enriched and labeled cells were fixed and permeabilized by Cytofix/Cytoperm™ Fixation/Permeabilization Solution Kit (BD, San Jose, CA), then stained with PE-conjugated anti Ki67 according to the standard procedure. BrdU^+^ or Ki67^+^ cells in TAMs or TAM subpopulations were analyzed by flow cytometry.

### Immunofluorescence and Wright-Giemsa staining

To identify Namalwa-V, Namalwa-M, and Namalwa-S, cells were collected and incubated with primary antibody against human M-CSF at a dilution of 1:100 followed by incubation with Dylight^TM^ 649-conjugated anti-mouse IgG before performed FACS or dropped on slides. To study the distribution of TAMs in tumor tissues, immunofluorescence staining of tumor sections (4-μm thick) was performed as described previously [31]. Briefly, sections were incubated with primary antibody against F4/80 at a dilution of 1:100 followed by incubation with Dylight^TM^ 649-conjugated anti-mouse IgG. Slides or sections were scanned analyzed under a confocal laser microscope (UltraView Vox, PerkinElmer, MA).

TAM subpopulations were sorted and spun to slides and Wright-Giemsa staining was performed. The slides were examined under a light microscope (AXIO Observer A1, ZEISS, Germany).

### Responses of TAMs to LPS and IL-4

TAMs were sorted from different tumor microenvironments and cultured in 24-well plates for 24hrs with or without 100 ng/ml LPS (Sigma-Aldrich, St. Louis) or 20ng/ml IL-4 (PeproTech, Rocky Hill, NJ).

### Real-time reverse transcription PCR

Total RNA was extracted with RNeasy mini Kit (Qiagen, Valencia, CA) according to the manufacture's instructions. Reverse transcription was achieved using Super Script First-Strand Synthesis System (Invitrogen, Carlsbad, CA). Real-time PCR was performed using a StepOnePlus Real-Time PCR System (AppliedBiosystems, Foster City, CA). The sequences for all primers are listed in Table S1.

### Nanopro immunoassay

Nanopro immunoassay (NIA) was performed using Nanopro 1000 (Protein Simple, Santa Clara, CA) following manufacturer's protocol. TAMs were sorted and lysed with Bicine/CHAPS Lysis buffer plus 1×DMSO inhibitor mix and 1×aqueous inhibitor mix (Protein Simple, Santa Clara, CA) at 10^4^ cells/μl. Cell lysis was mixed with 2.7M urea/53mM DTT (1:3) and treated for 5 min at RT. Then samples were mixed with Premix G2 5-8 (Protein Simple, Santa Clara, CA), including pI standard (Protein Simple, Santa Clara, CA) at a ratio of 1:3, and loaded into 384-well microplate. For each experiment, samples were analyzed in duplicate. Data analysis was carried out using Compass Software (Protein Simple, Santa Clara, CA).

### Dynamic array analysis

Dynamic array analysis of gene expression in TAM subpopulations was performed following instructions of BioMark™ real time PCR system. Briefly, 10^4^ cells were sorted directly into lysis buffer RLT (RNeasy mini-kit, Qiagen, Valencia, CA). RNA was extracted and reverse transcribed into cDNA for further specific target amplication (STA). STA consists of 2 min at 95°C to inactivate reverse transcriptase and activate Taq enzyme, followed by 14 cycles of 15 s at 95°C and 4 min at 60°C. Preamplified cDNA was diluted with TE buffer (1:5) before used for real-time PCR. Gene expression was analyzed using BioMark™ 96·96 Dynamic Array (Fluidigm, South San Francisco, CA). The PCR consists of 10 min hot-start at 95°C to activate the Taq polymerase, followed by a 40 cycles two-step program (15 s at 95°C and 60 s at 60°C). All The TaqMan primers and probes (AppliedBiosystems, LifeTechnologies, Foster City, CA) used were listed on table S2. Data were analyzed using BioMark™ Real-Time PCR Analysis Software (Fluidigm, South San Francisco, CA).

### Statistical analysis

The results were represented as means ± SD. Analysis was done using GraphPad Prism 6.0 software. Significance was determined by one-way or two-way analysis of AVONA. *P* < 0.05 was considered statistically significant.

## SUPPLEMENTARY MATERIAL FIGURES AND TABLES


