# Supplementary material for: Diverse in vivo effects of soluble and membrane-bound M-CSF on tumor-associated macrophages in lymphoma xenograft model
Source: Oncotarget. 2015 Nov 22;7(2):1354–66. doi: 10.18632/oncotarget.6362 (PMC4811465; doi:10.18632/oncotarget.6362)
Supplement: Supplementary file 1 [file oncotarget-07-1354-s001.pdf]

Diverse *in vivo* effects of soluble and membrane-bound M-CSF on tumor-associated macrophages in lymphoma xenograft model

Supplementary Material

Table S1 Primers used for detection of mouse genes by real time PCR analysis.

Names	Forward (5'-3')	Reverse (5'-3')
GAPDH	CACTTGAAGGGTGGAGC	GGGCTAAGCAGTTGGTG
CXCL11	AGCTGCTCAAG GCTTCCTTA	AGTAACAATCACTTCAACTTTGTCG
CXCL9	AGTGTGGAGTT CGAGGAACC	GAGTCCGGATC TAGGCAGG
iNOS	CCACCTCTATC AGGAAGAAA	CTGCACCGAAG ATATCTTCA
IL-1β	TGCCACCTTTT GACAGTGAT	TGTCCTCATCC TGGAAGGTC
IL-12	GCCAGGTGTCT TAGCCAGTC	AGCTCCCTCTT GTTGTGGAA
IL-6	CCGCTATGAAGTTCCTCTCTGC	ATCCTCTGTGAAGTCTCCTCTCC
Arg1	CAACCAGCTCTGGGAATCTG	AATCGGCCTTT TCTTCCTTC
CCL17	TGCTTCTGGGG ACTTTTCTG	TGGCCTTCTTC ACATGTTTG
CCL22	GTCCTTCTTGC TGTGGCAAT	ACGGTTATCAA AACAACGCC
CD206	CCTGAACAGCA ACTTGACCA	GCAATGGCCAT AGAAAGGAA
IL-10	CCAGAGCCACA TGCTCCTA	AGGGGAGAAAT CGATGACAG
M-CSF	TCACAACCTCA TCCTTCTGCG	GACCCAGTTAG TGCCCAGTGA
MCSFR	GCCTCATCCTCACGGTCCAT	TCAGATTATTCCAGCCTGCCTTGT
MMP9	TGAGTCCGGCA GACAATCCT	CCCTGGATCTC AGCAATAGCA
TNFα	AAGCCTGTAGCCCACGTCGTA	GGCACCACTAGTTGGTTGTCTTTG
VEGFα	TCCTCCTATCTCCACCACCTATCC	CTCACCGCCTTGGCTTGTC

Table S2 TaqMan primer and probes used in dynamic array analysis.

Genes	Species	Probe number
GAPDH	Mus musculus	Mm99999915_g1
IL-1β	Mus musculus	Mm00434228_m1
IL-12	Mus musculus	Mm00434165_m1
iNOS	Mus musculus	Mm00440502_m1
IL-6	Mus musculus	Mm00446190_m1
TNFα	Mus musculus	Mm00443258_m1
CXCL9	Mus musculus	Mm00434946_m1
CXCL11	Mus musculus	Mm00444662_m1
ARG1	Mus musculus	Mm00475988_m1
IL-10	Mus musculus	Mm00439614_m1
CD206	Mus musculus	Mm00485148_m1
UPA	Mus musculus	Mm01274460_g1
CXCL4	Mus musculus	Mm00451315_g1
TGFβ	Mus musculus	Mm01178820_m1
VEGFα	Mus musculus	Mm01281449_m1
M-CSF	Mus musculus	Mm00432686_m1
MMP9	Mus musculus	Mm00442991_m1
CCL17	Mus musculus	Mm01244826_g1
CCL22	Mus musculus	Mm00436439_m1

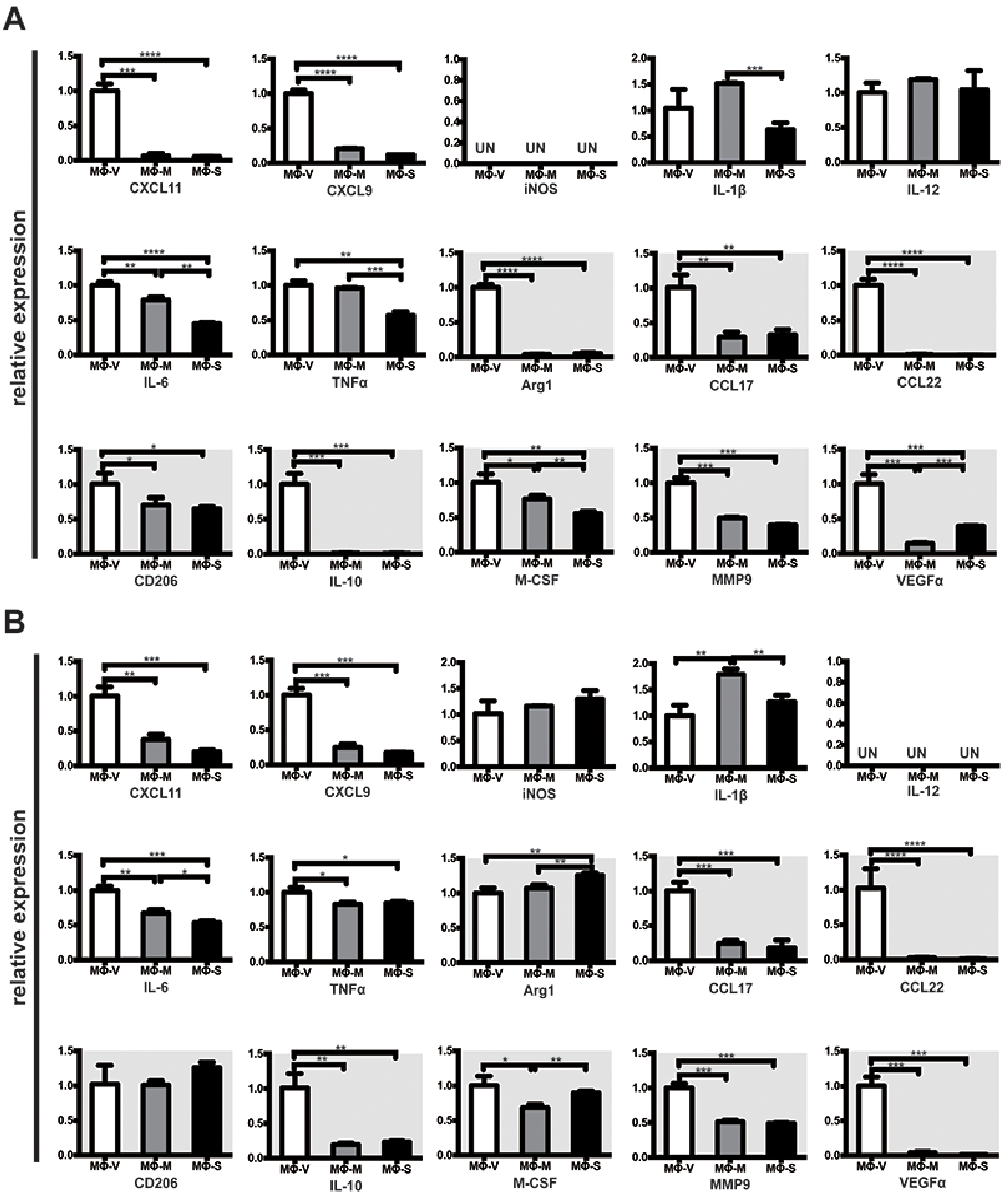

Fig S1. **Expression of phenotype-associated genes in macrophages from bone marrow and peritoneal of tumor bearing mice.** Macrophages were isolated from bone marrow (A) or peritoneal (B) of tumor-bearing mice and the expression of phenotype-associated genes was detected by real time PCR. For each gene, the RQ value of Mφ-V was designated 1.000, respectively. The M2-related genes were shown with dark background. UN=undetected. Data are shown as mean ± SD (n=3, *, $p<0.05$; **, $p<0.01$; ***, $p<0.001$; ****, $p<0.0001$).

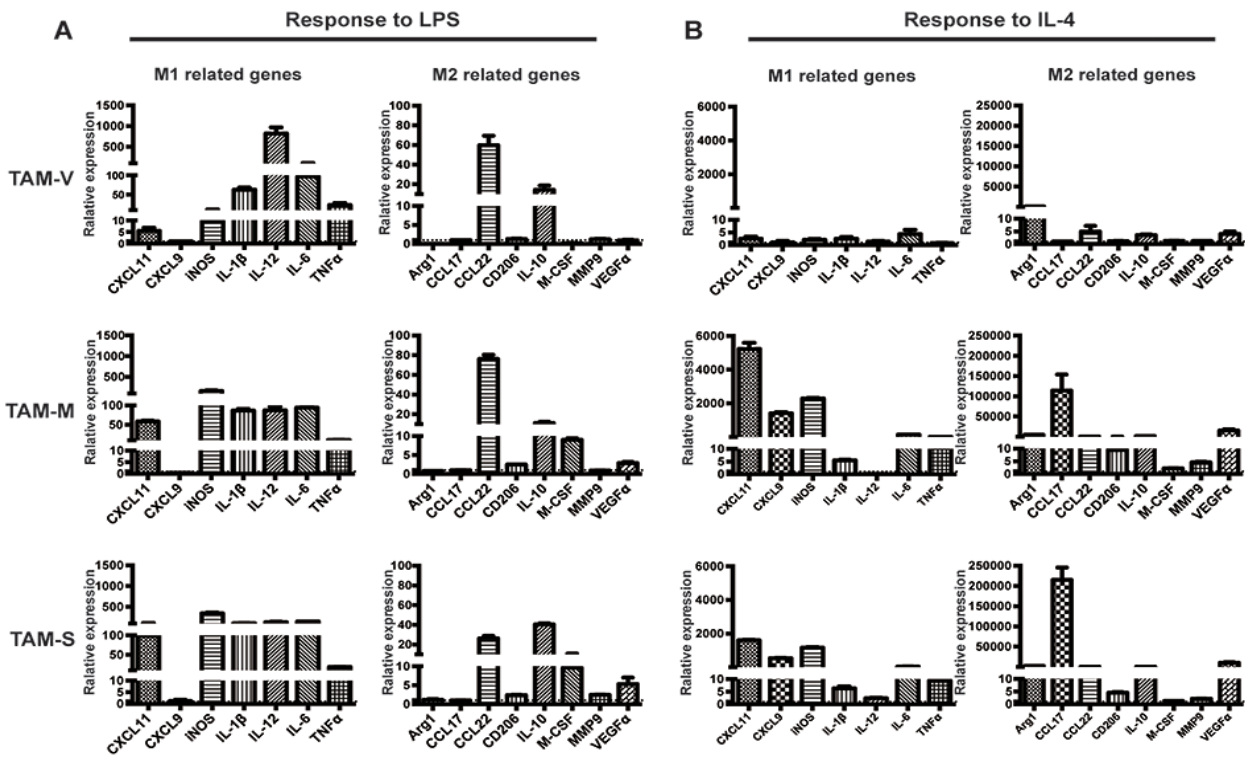

Fig S2. **Responses of TAMs to LPS and IL-4.** Single cell suspension of tumor tissues on Day 30~35 was obtained and TAMs were sorted by flow cytometer after enrichment by magnetic beads. TAMs were cultured in 24-well plates and treated with LPS (A) or IL-4 (B) for 24hrs. The expression of phenotype-associated genes was detected by real time PCR (n=3). For TAMs from different tumor microenvironments, the RQ value of gene expression in untreated TAM-V was designated 1.000.
